# Analysis of infections among patients with historical culture positive for extended-spectrum beta-lactamase (ESBL)–producing *Escherichia coli* or *Klebsiella pneumoniae*: Is ESBL-targeted therapy always needed?

**DOI:** 10.1017/ash.2022.363

**Published:** 2023-03-08

**Authors:** Tyler J. Stone, Michael DeWitt, James W. Johnson, James R. Beardsley, Iqra Munawar, Elizabeth Palavecino, Vera P. Luther, Christopher A. Ohl, John C. Williamson

**Affiliations:** 1 Department of Pharmacy, Atrium Health Wake Forest Baptist, Winston-Salem, North Carolina; 2 Section on Infectious Diseases, Department of Internal Medicine, Wake Forest University School of Medicine, Winston-Salem, North Carolina; 3 Department of Pathology, Atrium Health Wake Forest Baptist, Winston-Salem, North Carolina

## Abstract

**Objective:**

Among patients with a history of ESBL infection, uncertainty remains regarding whether all of these patients require ESBL-targeted therapy when presenting with a subsequent infection. We sought to determine the risks associated with a subsequent ESBL infection to help inform empiric antibiotic decisions.

**Methods:**

A retrospective cohort study of adult patients with positive index culture for *Escherichia coli* or *Klebsiella pneumoniae* (EC/KP) receiving medical care during 2017 was conducted. Risk assessments were performed to identify factors associated with subsequent infection caused by ESBL-producing EC/KP.

**Results:**

In total, 200 patients were included in the cohort, 100 with ESBL-producing EC/KP and 100 with ESBL-negative EC/KP. Of 100 patients (50%) who developed a subsequent infection, 22 infections were ESBL-producing EC/KP, 43 were other bacteria, and 35 had no or negative cultures. Subsequent infection caused by ESBL-producing EC/KP only occurred when the index culture was also ESBL-producing (22 vs 0). Among those with ESBL-producing index culture, the incidences of subsequent infection caused by ESBL-producing EC/KP versus other bacterial subsequent infection were similar (22 vs 18; *P* = .428). Factors associated with subsequent infection caused by ESBL-producing EC/KP include history of ESBL-producing index culture, time ≤180 days between index culture and subsequent infection, male sex, and Charlson comorbidity index score >3.

**Conclusions:**

History of ESBL-producing EC/KP culture is associated with subsequent infection caused by ESBL-producing EC/KP, particularly within 180 days after the historical culture. Among patients presenting with infection and a history of ESBL-producing EC/KP, other factors should be considered in making empiric antibiotic decisions, and ESBL-targeted therapy may not always be warranted.

Extended-spectrum β-lactamase (ESBL) enzymes are characterized as a clinically relevant resistance mechanism produced by gram-negative bacteria. Enterobacterales, such as *Klebsiella pneumoniae* and *Escherichia coli,* are common gram-negative bacterial pathogens that produce ESBLs.^1^ ESBL-producing *Enterobacterales* confer resistance to third-generation cephalosporins, extended-spectrum penicillins, and aztreonam.^2^ An increasing amount of ESBL-producing bacteria are also associated with resistance to other antimicrobial classes such as fluoroquinolones, aminoglycosides, and tetracyclines.^3^ In 2019, the US Centers for Disease Control and Prevention (CDC) reported that ESBL-producing Enterobacterales are associated with nearly 197,000 healthcare-associated infections and 9,100 deaths each year.^4^ In a recent study of meropenem versus piperacillin-tazobactam in the treatment of bloodstream infections caused by ceftriaxone nonsusceptible *K. pneumoniae* or *E. coli*, noninferiority of 30-day mortality was not achieved, with results favoring meropenem.^5^ For these reasons, infections caused by ESBL-producing Enterobacterales are increasingly difficult to treat empirically. They contribute to the use of broad-spectrum antibiotics, and they are considered a national and global health concern.^3^

Prior studies have identified risk factors associated with infection caused by ESBL-producing Enterobacterales, such as major surgery, previous antimicrobial exposure, indwelling catheters, intubation or tracheostomy, prolonged hospital or intensive care unit (ICU) stays, residency in long-term care facilities, and cancer.^6–9^ Once a patient is infected with an ESBL-producing Enterobacterales, the likelihood of subsequent infection caused by ESBL-producing Enterobacterales is unknown, although history of antibiotic resistance has been shown to confer risk of the same antibiotic resistance in subsequent infections.^10^ If a patient has a history of ESBL-producing EC/KP, empiric therapy with a carbapenem has become common practice in hospitals. With rising rates of infection caused by ESBL-producers, there is concern that overuse of carbapenems will contribute to escalating rates of resistance.^11^ In this study, we evaluated the microbiology of infections among patients with a history of ESBL-producing EC/KP. Specifically, we sought to determine the likelihood of having a subsequent infection caused by ESBL-producing *E. coli* or *K. pneumoniae*.

## Methods

This retrospective cohort study was conducted at Wake Forest Baptist Health (WFBH), a health system that includes an 885-bed, academic, tertiary-care hospital in Winston-Salem, North Carolina, 4 affiliated community hospitals, and a network of clinics. Inpatients and outpatients at least 18 years of age receiving care within the WFBH system and whose electronic medical record (EMR) included a clinical specimen culture positive for ESBL-producing *E. coli* and/or *K. pneumoniae* (EC/KP) during calendar year 2017 were eligible. These patients were randomly selected and evaluated for inclusion from a report generated by the EMR software up to 100 patients that met criteria. For analysis purposes, an equal number (100) of patients were identified who had a clinical specimen culture in 2017 positive for EC/KP and negative for ESBL production. These cultures were considered the index cultures for these patients.

Patients were excluded if they had a culture positive with EC/KP within 1 year prior to the index culture, regardless of ESBL result. Each patient was included only once. If a patient had EC/KP isolated on multiple occasions in 2017, the first episode in the calendar year was deemed the index culture. Data were collected by review of the EMR for a 1-year follow-up period after the date of index culture, including information about any subsequent infections, antibiotics administered, demographic information, immune status, Charlson comorbidity index score, and laboratory information, including culture and susceptibility data. Subsequent infections were considered clinically significant and included in the analysis only if the treating provider prescribed an antibiotic. Patients with index cultures positive for ESBL-producing EC/KP were compared to those with index culture positive for EC/KP but negative for ESBL.

Comparisons for categorical data were performed using the χ^2^ or Fisher exact test. The Mann-Whitney U test or Student *t* test was utilized for ordinal and continuous data, depending on the distribution. A 2-sided *P* ≤ .05 was considered statistically significant. In univariate analysis, patients with subsequent infection caused by ESBL-producing EC/KP were compared to all other patients, a combined group of those with subsequent infection caused by a different pathogen and those without subsequent infection. Factors analyzed included age, sex, Charlson comorbidity index score, time from index culture to subsequent infection, and antibiotics received in the 90 days before subsequent infection. In analyzing time from index culture to subsequent infection, patients without subsequent infection were censored at the end of the observation period (365 days).

Multivariable logistic regression analysis was performed to determine risk factors independently associated with subsequent infection caused by ESBL-producing EC/KP. To assess the likelihood of subsequent infection caused by ESBL-producing EC/KP among those with ESBL-producing index culture, a logistic model was fit using maximum likelihood. Restricted cubic splines were used to relax the linearity assumptions on the effect of time. Covariates were selected from a prespecified list to optimize the c-index of the model, and 500 bootstrap samples were used for model validation and calibration. Statistical analyses were performed using R software version 4.0.2 software.^12^

To further explore the risk of developing a subsequent infection caused by ESBL-producing EC/KP, we performed time-to-event analyses. An event was defined as subsequent infection caused by ESBL-producing EC/KP. Study participants were censored at day 365 if no subsequent infection caused by ESBL-producing EC/KP occurred. To investigate the unadjusted incidence of subsequent infection caused by ESBL-producing EC/KP, we utilized a Kaplan-Meier approach, and a log-rank test was performed to assess differences between the groups.

## Results

**Fig. 1.**
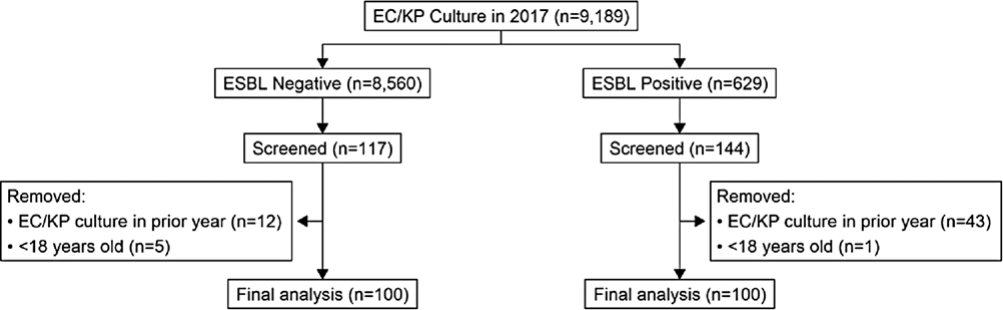
Flow diagram of subject selection

In total, 8,560 patients with a culture containing ESBL-negative EC/KP in 2017 and 629 patients with a culture containing ESBL-producing EC/KP in 2017 were initially identified. Furthermore, 144 of the 629 patients with ESBL-producing EC/KP, and 117 of the 8,560 patients with ESBL-negative EC/KP underwent screening for inclusion. However, 43 patients with cultures positive for ESBL-producing EC/KP and 12 patients with ESBL-negative cultures were excluded due to having a positive EC/KP culture within the prior year. Also, 1 patient with ESBL-positive culture and 5 patients with ESBL-negative culture were excluded due to age <18 years at the time of specimen collection, leaving 200 patients selected for the study (Fig. 1).

The mean patient age was 58 years, 84% were female, and 69% were outpatients. Also, 86% of index cultures were *E. coli* and 86% were from urine specimens. Table 1 provides patient characteristics within groups based on ESBL test result of the index culture. Although no characteristics were statistically different between these groups, those with ESBL-producing EC/KP index cultures were generally older (63.1 vs 55.5 years) and had a greater burden of comorbidities (mean Charlson comorbidity index score, 4.3 vs 2.6).

**Table 1.** Patient Characteristics

Characteristic	Patients With ESBL-Producing EC/KP Index Culture (n=100), No. (%)^a^	Patients With ESBL-negative EC/KP Index Culture (n=100), No. (%)^a^
Sex, female	78 (78)	89 (89)
Age, mean y (SD)	63.1 (18.5)	55.5 (21.6)
Outpatient	58 (58)	80 (80)
**Index culture site**		
Urine	79 (79)	93 (93)
Sputum	7 (7)	3 (3)
Blood	7 (7)	1 (1)
Wound	4 (4)	2 (2)
Other	3 (3)	1 (1)
**Index culture**		
*Escherichia coli*	83 (83)	88 (88)
*Klebsiella pneumoniae*	17 (17)	12 (12)
Charlson comorbidity index score, mean (SD)	4.3 (3.2)	2.6 (2.9)
Immunocompromised	7 (7)	1 (1)

Note. ESBL, extended-spectrum β-lactamase; EC/KC, *E. coli* or *K. pneumoniae*.

a Units unless indicated otherwise.

Within 1 year of index culture, 100 patients (50%) developed a subsequent infection; 65 subsequent infections were positive upon bacterial culture. Among them, 22 were caused by ESBL-producing EC/KP, 43 were caused by bacteria other than ESBL-producing EC/KP, and 35 had a negative culture or no culture was performed in association with the subsequent infection (eg, viral upper respiratory tract infection). The mean times from index culture to subsequent infection caused by ESBL-producing EC/KP versus those caused by a bacteria other than ESBL-producing EC/KP were 85 days (range, 26–226) and 140 days (range, 15–363), respectively (*P* = .014). A greater percentage of culture-positive subsequent infections caused by ESBL-producing EC/KP occurred within 180 days of index culture compared to subsequent infections not caused by ESBL-producing EC/KP: 21 (96%) of 22 versus 26 (60%) of 43, respectively (*P* = .003).

Table 2 describes culture-positive subsequent infections according to ESBL status of the index culture. Of the 65 culture-positive subsequent infections, 40 occurred among patients with ESBL-producing index culture and 25 occurred among patients with ESBL-negative index culture. subsequent infections caused by ESBL-producing EC/KP occurred exclusively in the group with ESBL-producing index cultures (100% vs 0%). Of the 43 culture-positive subsequent infections caused by organisms other than ESBL-producing EC/KP, 18 (43%) occurred among patients with ESBL-producing index culture and 25 (58%) occurred among patients with ESBL-negative index culture. Table 3 compares characteristics of patients with subsequent infection caused by ESBL-producing EC/KP to those caused by a bacteria other than ESBL-producing EC/KP. Subsequent infection caused by ESBL-producing EC/KP was associated with male sex and ESBL-producing index culture. Although not statistically significant, patients with subsequent infection caused by ESBL-producing EC/KP were older (67 vs 60 years; *P* = .091) and had higher average Charlson comorbidity index score (3.5 vs 2.6; *P* = .098). Additionally, the mean days between index culture and subsequent infection was significantly longer in patients whose bacterial subsequent infection was not ESBL-producing EC/KP: mean, 140 days (95% CI ±104) versus 85 days (95% CI, ±65; *P* = .014).

**Table 2.** Culture-Positive Subsequent Infections According to ESBL Status of Index Culture

Index Culture	Culture Positive Subsequent Infections^a^	
	ESBL-producing EC/KP (n=22), No. (%)	Other Bacteria (n=43), No. (%)^b^
ESBL-producing EC/KP (n=100)	22 (100)	18 (42)
ESBL-negative EC/KP (n=100)	0 (0)	25 (58)

Note. ESBL, extended-spectrum β-lactamase; EC/KC, *E. coli* or *K. pneumoniae*.

a Occurring within 1 year after index culture.

b Includes culture-positive subsequent infections caused by any bacteria other than ESBL-producing *E. coli* or *K. pneumoniae*.

**Table 3.** Characteristics of Patients with Culture-Positive Subsequent Infections

Factor	Culture-Positive Subsequent Infection		*P* Value
	ESBL-producing EC/KP (n=22)^a^	Other bacteria^1^ (n=43)^a,^ ^b^	
Age, mean y (SD)	67	60	.091
Sex, male	7 (32)	3 (7)	.009
Immunocompromised	0 (0)	3 (7)	.700
Charlson comorbidity index score, mean (SD)	3.5 (3.1)	2.6 (2.4)	.098
History of index culture caused by ESBL-producing EC/KP^2^	22 (100)	18 (42)	<.001
Days between index culture and subsequent infection, mean (SD)	85 (64.6)	140 (103.9)	.014
Antibiotics received in previous 90 d	17 (77)	25 (58)	.127

Note. ESBL, extended-spectrum β-lactamase; EC/KC, *E. coli* or *K. pneumoniae*.

a Data presented as no. (%) unless indicated otherwise.

b Includes culture-positive subsequent infections caused by any bacteria other than ESBL-producing *E. coli* or *K. pneumoniae.*

**Fig. 2.**
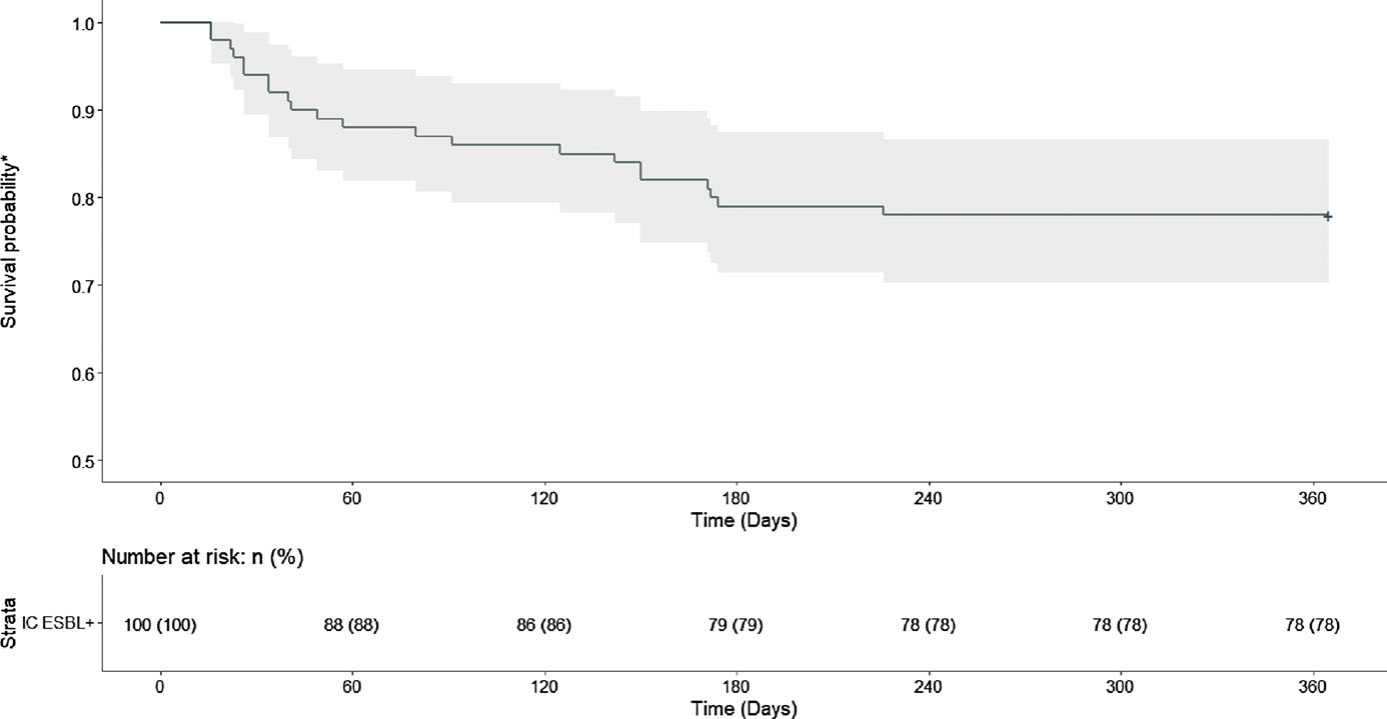
Time to Subsequent Infection Caused by ESBL-Producing *E. Coli* or *K. pneumonia* *Probability of remaining free of subsequent infection caused by ESBL-producing *E. Coli* or *K. pneumonia* IC = index culture

Figure 2 shows the Kaplan Meier curve of time to subsequent infection caused by ESBL-producing EC/KP. In the group with ESBL-producing index culture, the probability of remaining free of subsequent infection caused by ESBL-producing EC/KP declined within the first 180 days after index culture. Only 1 patient experienced ESBL-producing subsequent infection after 180 days, and by day 226, no additional subsequent infections caused by ESBL-producing EC/KP were observed, indicating a stable survival probability of 0.769 (95% CI, 0.689–0.859) after day 240. A log-rank test demonstrated statistical difference between the 2 groups (*P* < .0001).

The results of univariate and multivariable analyses are shown in Table 4. The strongest factors associated with subsequent infection caused by ESBL-producing EC/KP were having an ESBL-producing index culture and subsequent infection occurring ≤180 days after index culture. Index culture status was not a variable included in the univariate or multivariable analyses due to complete separation (100% predictability). Male sex was also associated with subsequent infection caused by ESBL-producing EC/KP. The point estimate for Charlson comorbidity index score >3 was consistent with a positive association. Although significant in univariate analysis, antibiotic exposure in the past 90 days was attenuated in the multivariable model. Based on results of these analyses, a diagnostic nomogram was developed for predicting the likelihood of subsequent infection caused by ESBL-producing EC/KP, including the effect of time as a continuous variable. To achieve higher predictive accuracy and maintain a more parsimonious model, sex was not included as a variable in the nomogram. The nomogram and instructions for use are provided in the in Supplementary Materials.

**Table 4.** Factors Associated with Subsequent Infection Caused by ESBL-Producing *Escherichia coli* or *Klebsiella pneumoniae* ^a^

			Univariate		Multivariable	
Characteristics	Overall (N = 200)^b^	Event (n = 22)^b^,^c^	Odds Ratio (95% CI)	*P* Value	Odds Ratio (95% CI)	*P* Value
**Age, mean y (IQR)**	62 (43–74)	70 (58– 76)	1.02 (1.00–1.05)	.052	0.99 (0.95–1.04)	.80
**Sex**						
Female	167 (84)	15 (68)				
Male	33 (16)	7 (32)	2.73 (0.96–7.16)	.047	6.19 (1.01–37.8)	.049
**Charlson comorbidity index score**						
≤ 3	106 (53)	4 (18)				
> 3	94 (47)	18 (82)	6.04 (2.15–21.6)	.002	7.10 (0.99–50.8)	.051
**Antibiotic exposure past 90 d**						
No	159 (80)	5 (23)				
Yes	41 (20)	17 (77)	21.8 (7.84–71.5)	<.001	2.15 (0.40–11.6)	.40
**Subsequent infection ≤180 d after index culture**						
No	153 (77)	1 (5)				
Yes	47 (23)	21 (95)	123 (24.1–2,248)	<.001	108 (8.27–1,401)	<.001

Note. ESBL, extended-spectrum β-lactamase; IQR, interquartile range; CI, confidence interval.

a Index culture ESBL-producing was not included in the multivariable model due to perfect separation (100% predictive).

b Data presented as no. (%) unless indicated otherwise.

c Subsequent infection caused by ESBL-producing *E. coli* or *K. pneumoniae*.

## Discussion

The incidence of ESBL-producing *E. coli* and *Klebsiella* is rising throughout the United States.^2^ Carbapenems are generally considered the drugs of choice for serious infections caused by ESBL-producing Enterobacterales.^14^ However, mounting utilization of carbapenems to treat infections caused by ESBL-producers has led to increased prevalence of carbapenem-resistant Enterobacterales in the United States and worldwide.^4,15^ Many institutions restrict the use of carbapenems out of concern for the development of carbapenem resistance. Preference at these institutions is given to other broad-spectrum antibiotics such as piperacillin-tazobactam and cefepime. Yet, these antibiotics are not reliable as treatment of serious infections caused by ESBL-producing EC/KP, particularly bloodstream infections.^5^ It is imperative to describe what factors are important in determining which patients are most likely to have infection caused by ESBL-producing EC/KP so that optimal outcomes are preserved by appropriately selecting a carbapenem as empiric treatment, even if restrictions are in place, while minimizing unnecessary use of carbapenems to delay the development of resistance. This study has identified the factors that clinicians can use in making these difficult decisions about who should receive an empiric carbapenem.

Prior infection with ESBL-producing EC/KP was a strong predictor of ESBL infection in our study. Certainly, it is considered best practice to review prior cultures and history of resistant organisms when deciding on empiric antibiotic therapy for patients presenting with signs and symptoms of infection.^10,16^ However, we identified a timing effect that influenced the likelihood of having infection caused by ESBL-producing EC/KP. Specifically, the risk of having ESBL-producing EC/KP was significantly reduced if the historical infection was >180 days prior. Based on this information, it is reasonable to consider noncarbapenem antibiotics when choosing empiric therapy for those patients whose historical infection was >180 days prior. Male sex and Charlson comorbidity index scores >3 were also positively associated with infection by ESBL-producing EC/KP. The latter is not surprising because other studies have recognized the impact of comorbid conditions on risk of infection caused by resistant organisms.^17^ As such, empiric carbapenem therapy may be more appropriate for medically complex patients with multiple underlying disease states but particularly if the patient also has history of ESBL-producing EC/KP within the past 180 days.

Why the risk of infection caused by ESBL-producing EC/KP decreases over time among patients with a prior ESBL-producing EC/KP culture is not obvious, but colonization may be an important contributor to this trend. In a study by Birgand et al,^18^ colonization with an ESBL-producing organism did not persist indefinitely. In fact, this study demonstrated a median duration of colonization that closely aligns with the 180-day time course identified in our study.^18^ Among patients with bloodstream infection, researchers have noted a steadily declining trend in the positive predictive value of historical, nonsurveillance ESBL cultures as time increases since the historical culture.^10^ It stands to reason that colonization with an ESBL-producing organism would precede infection with that organism and that any change in colonization status over time would result in a change in risk of infection by that organism.

Indiscriminate use of carbapenems is associated with higher rates of carbapenem resistance among gram-negative bacteria.^19,20^ Additionally, inappropriate use of broad-spectrum antibiotics, such as carbapenems, leads to excess risk of *C. difficile* infections.^21^ For these reasons, many antimicrobial stewardship programs at hospitals either develop guidelines to direct the use of carbapenems or they limit use via prior authorization (restriction) processes. Results from this study have compelling implications for how hospital providers and stewardship team members approach clinical decision making regarding empiric carbapenems. It is not sufficient to say that all patients without a history of ESBL-producing EC/KP should not receive an empiric carbapenem. Indeed, we did not assess the risk of having an ESBL-producing EC/KP among patients presenting with their first EC/KP infection. Furthermore, as the incidence of ESBL-positivity among EC/KP continues to trend upward, the risk of having a first EC/KP infection that is ESBL-producing will also rise. However, results of this study add to our knowledge of patient-specific factors that influence risk of infection caused by ESBL-producing EC/KP. Clinicians may find these results useful as another tool in deciding which patients are most appropriate to receive an empiric carbapenem, ultimately with the goal of preserving the future utility of carbapenems, vis-à-vis preventing carbapenem resistance from developing.

This study had several limitations. Although ESBL-producing bacteria are commonly isolated from the genitourinary tract, this study had relatively low representation of isolates from nonurinary sources. Future studies should aim to determine whether the findings of this study are applicable across all anatomic sources of infection because this may be a factor contributing to clinical outcomes.^22^ Similarly, this study included a significant proportion of outpatients. A health-system stewardship worker may not feel completely comfortable extrapolating these results to their patient population. However, a separate study analyzing usefulness of prior cultures to predict resistance among hospitalized patients with bloodstream infection documented declining positive predictive value over time since historical culture.^10^ The results of this study may not be applicable to locations where the rate of ESBL-producing EC/KP is significantly different from that of WFBH. In addition, we analyzed ESBL-producing *E. coli* and *K. pneumoniae* collectively and exclusive of other resistant bacteria. It is unknown whether our findings have implications for the management of other bacteria harboring ESBL enzymes or whether there is a difference specifically between ESBL-producing *E. coli* and ESBL-producing *K. pneumoniae*. Access to medical records outside of the WFBH system was limited. Therefore, it is possible that a patient had an infection and received treatment that was not reflected in the data collected. Lastly, the retrospective nature of this study relies on accuracy of documentation in the EMR.

In conclusion, history of positive culture for ESBL-producing EC/KP is associated with subsequent infection caused by ESBL-producing EC/KP. However, the results of this study demonstrate that other factors should weigh in the decision to use an empiric carbapenem for these patients. Patients presenting >6 months after historical culture with ESBL-producing EC/KP have substantially lower risk of infection caused by ESBL-producing EC/KP. For this reason, clinicians and stewardship workers should consider noncarbapenem antibiotics for empiric therapy of these non–life-threatening subsequent infections. Other factors, such as male sex, comorbidities, and severity of illness should also be considered when making antibiotic decisions. Nonetheless, the categorical use of empiric carbapenems for all patients with historical cultures of ESBL-producing EC/KP is not warranted. An approach to choosing empiric antibiotic therapy in these patients based on the factors identified in this study serves as another avenue to limit the development of resistant bacteria.

## Supplementary material

For supplementary material accompanying this paper visit http://doi.org/10.1017/ash.2022.363. click here to view supplementary material
