# Supplementary material for: Analysis of infections among patients with historical culture positive for extended-spectrum beta-lactamase (ESBL)–producing Escherichia coli or Klebsiella pneumoniae: Is ESBL-targeted therapy always needed?
Source: Antimicrob Steward Healthc Epidemiol. 2023 Mar 8;3(1):e47. doi: 10.1017/ash.2022.363 (PMC10031583; doi:10.1017/ash.2022.363)
Supplement: Supplementary file 1 [file S2732494X22003631sup001.docx]

Supplemental Material

Nomogram to Predict Probability of Subsequent Infection Caused by ESBL-Positive *E. coli* or *K. pneumoniae*


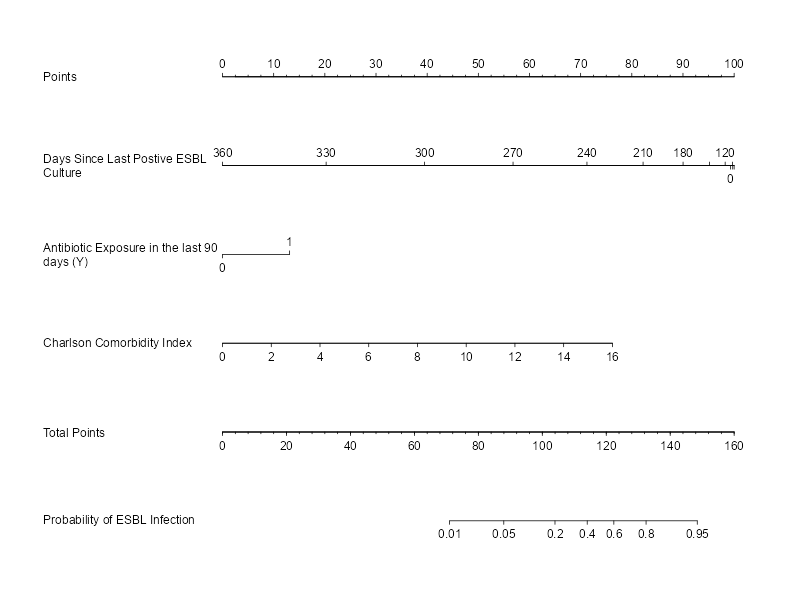


Use of this nomogram is intended for patients who present with suspected bacterial infection and have a history of culture growing ESBL-positive *E. coli* or *K. pneumoniae*.

Nomogram instructions:

1. The top line (Points) is to be used to assign points to the three variables below it.
2. Variable 1: Determine time in days since historical culture growing ESBL-positive *E. coli* or *K. pneumoniae*. Assign points based on this result using ‘Points’ line above.
3. Variable 2: Determine if the patient has received an antibiotic in the previous 90 days. If no, assign 0 points. If yes, assign 12 points.
4. Variable 3: Calculate the patient’s Charlson Comorbidity Index score. Assign points based on this result using the ‘Points’ line above.
5. Sum the assigned points for the three variables, and mark the result on the ‘Total Points’ line.
6. Match the mark on the Total Points line with the location on the line below. This is the probability the infection is caused by ESBL-positive *E. coli* or *K. pneumoniae*.

An online calculator for this nomogram may be found at www.ESBLrisk.com.
